# CaCu_1.424_Fe_0.576_Si_2_

**DOI:** 10.1107/S2414314625003256

**Published:** 2025-04-17

**Authors:** Yangkun Dai, Yibo Liu, Marek Mihalkovic, Bin Wen, Lifeng Zhang, Changzeng Fan

**Affiliations:** a State Key Laboratory of Metastable Materials Science and Technology Yanshan University,Qinhuangdao 066004 People’s Republic of China; b Institute of Physics, Slovak Academy of Sciences, 84511 Bratislava, Slovakia; c School of Mechanical and Materials Engineering North China University of Technology,Beijing 100144 People’s Republic of China; d Hebei Key Lab for Optimizing Metal Product Technology and Performance Yanshan University,Qinhuangdao 066004 People’s Republic of China; Goethe-Universität Frankfurt, Germany

**Keywords:** crystal structure, high-pressure sinter­ing, inter­metallic, Cu-Fe co-occupation

## Abstract

A CaCu_1.424_Fe_0.576_Si_2_ phase was obtained during high-pressure sinter­ing of an Si-rich quasicrystal composition prealloy with the nominal chemical com­position Si_61_Cu_30_Ca_7_Fe_2_. The obtained phase crystallizes in the space group *I*4/*mmm* (No. 139), with *a* = *b* = 4.041 Å and *c* = 10.010 Å. It is isotypic with CaCu_2_Si_2_ (*a* = *b* = 4.06 Å and *c* = 9.91 Å) [Palenzona *et al.* (1986 ▸). *J. Less-Common Met.***119**, 199–209] and CaFe_2_Si_2_ (*a* = *b* = 3.94 Å and *c* = 10.19 Å) [Hlukhyy *et al.* (2012 ▸). *Z. Anorg. Allg. Chem.***638**, 1619–1619]. It features a co-occupancy of Cu and Fe atoms with a ratio of the refined site-occupancy factors of 0.71 (15):0.29 (15).

## Abstract 2

A CaCu_1.424_Fe_0.576_Si_2_ phase was obtained during high-pressure sinter­ing of an Si-rich quasicrystal composition prealloy with the nominal chemical com­position Si_61_Cu_30_Ca_7_Fe_2_. The obtained phase crystallizes in the space group *I*4/*mmm* (No. 139), with *a* = *b* = 4.041 Å and *c* = 10.010 Å.

## Graphical abstract

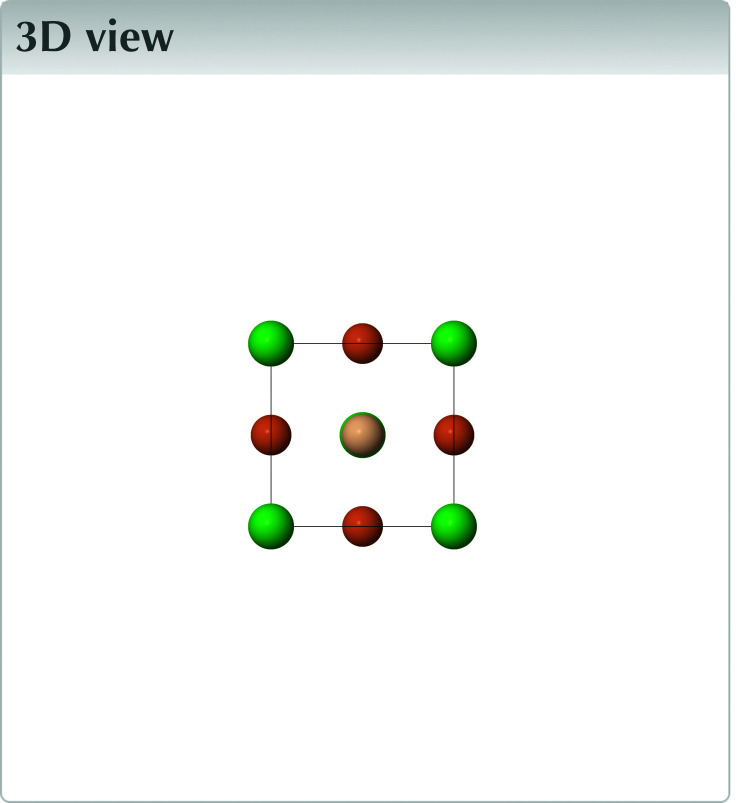

## Structure description

It has been reported that Si-rich quasicrystals form under extreme conditions during atomic bomb explosion (Bindi *et al.*, 2021 ▸). In this work, we took the Si-rich quasicrystal compostion and applied our high-pressure sintering methodology to reveal phases forming at this composition in a laboratory experiment and obtained crystals of the com­position CaCu_1.424_Fe_0.576_Si_2_. This phase shows remarkable structural similarities to BaFe_1.8_Co_0.2_As_2_ (*a* = *b* = 3.96 Å and *c* = 13.96 Å) reported by Sefat *et al.* (2008 ▸), sharing identical space-group symmetry and analogous co-site-occupation behaviour. CaCu_1.424_Fe_0.576_Si_2_, as well as BaFe_1.8_Co_0.2_As_2_, and along with other AETX-type com­pounds (AE = alkaline earth metals, T = transition metals and X = Si, Ge, As), belong to the 122-type structure and all show the space group *I*4/*mmm*.

**Figure 1:**
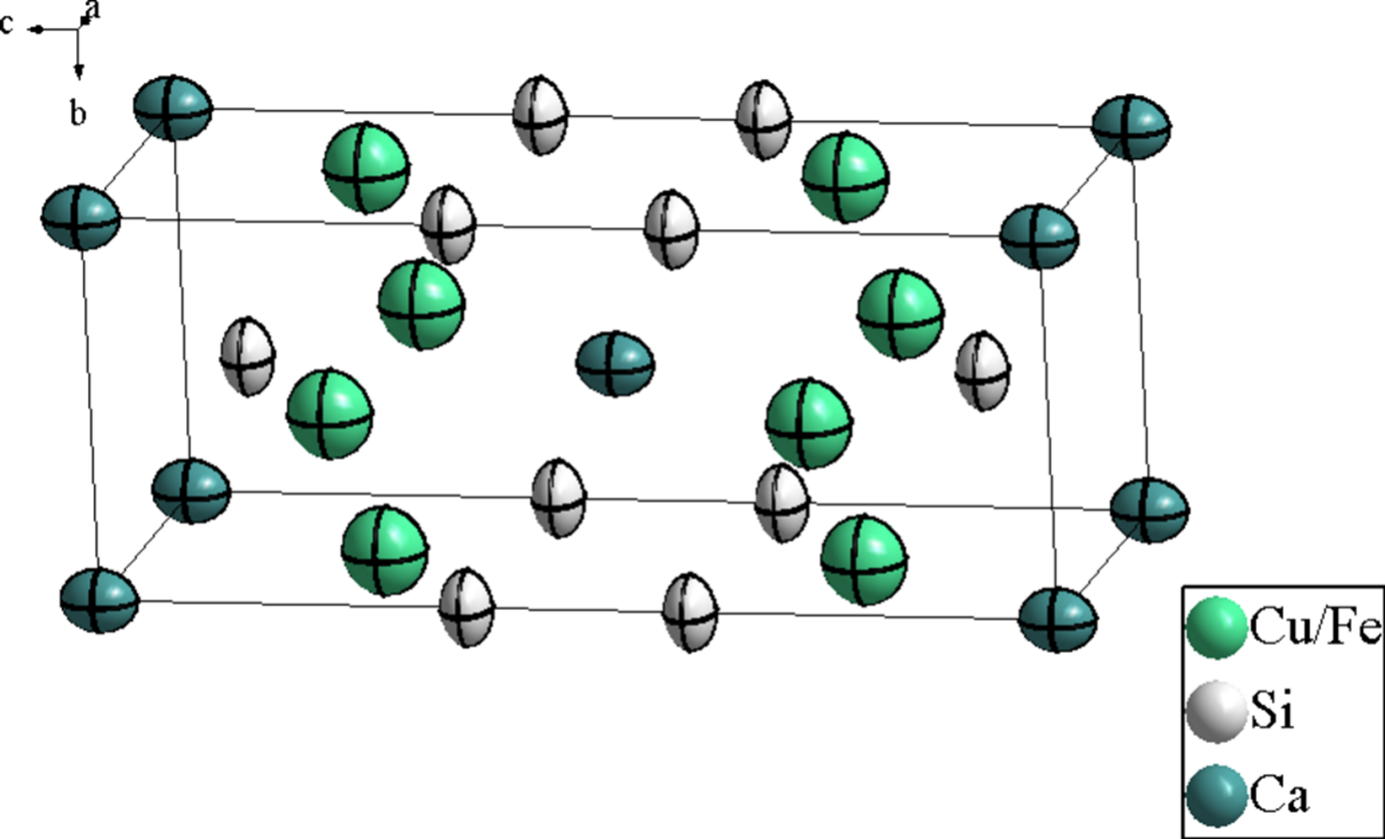
The crystal structure of CaCu_1.424_Fe_0.576_Si_2_ (one unit cell), with displacement ellipsoids drawn at the 99% probability level.

**Figure 2:**
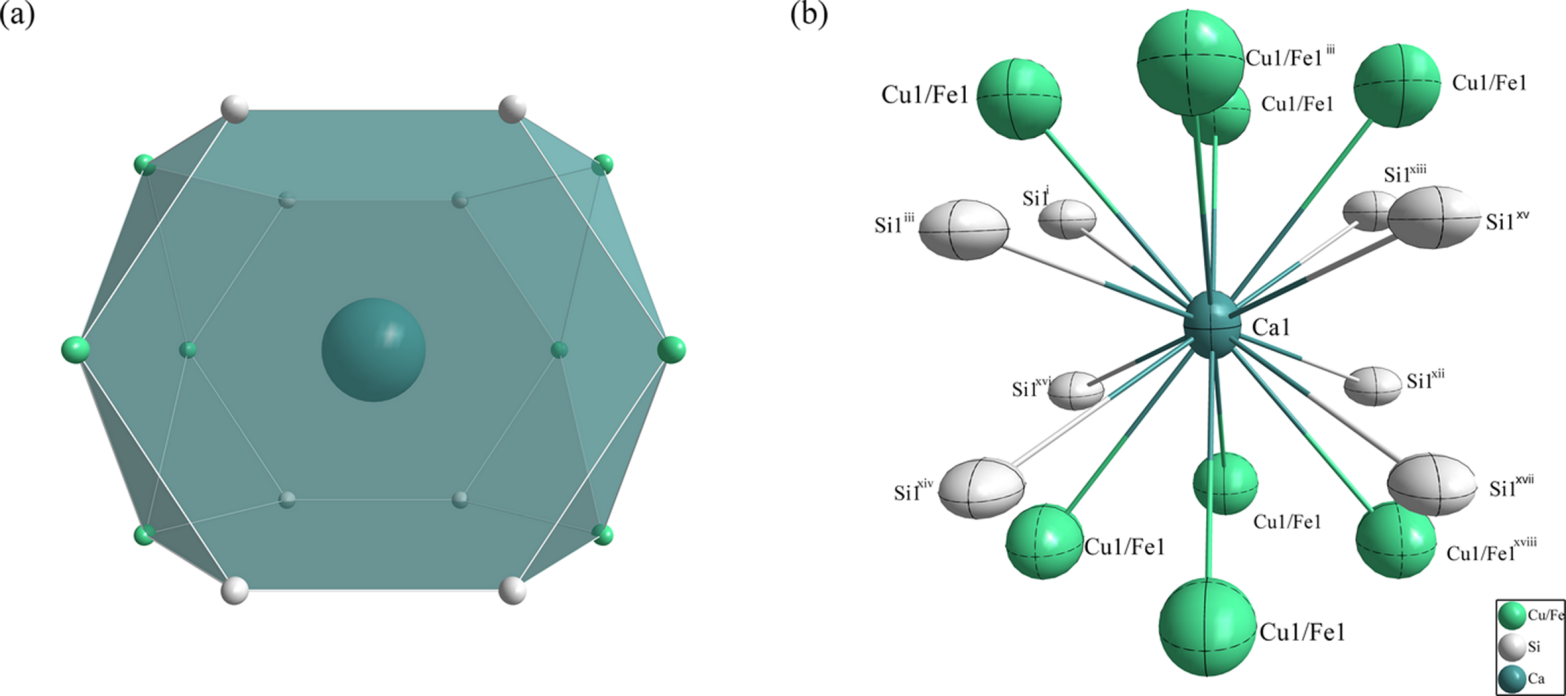
(*a*) The tetra­deca­hedron formed around the Ca1 atom at the 2 *a* site and (*b*) the environment of the Ca1 atom, with displacement ellipsoids given at the 99% probability level. [Symmetry codes: (i) −*x* − 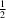, −*y* + 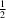, −*z* + 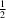; (iii) −*x* + 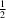, −*y* + 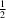, −*z* + 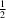; (xii) *x* − 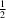, *y* − 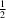, *z* − 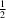; (xiii) −*x* − 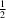, −*y* − 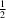, −*z* + 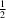; (xiv) *x* + 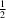, *y* + 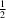, *z* − 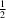; (xv) −*x* + 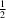, −*y* − 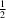, −*z* + 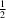; (xvi) *x* − 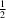, *y* + 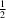, *z* − 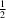; (xvii) *x* + 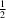, *y* − 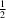, *z* − 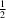; (xviii) −*x*, −*y*, −*z*.]

The distribution of atoms in the crystal unit of CaCu_1.424_Fe_0.576_Si_2_ is illustrated in Fig. 1 ▸. The coordination environment of the Ca atom is shown in Fig. 2 ▸. The Ca1 atom is located in a position with 4/*mmm* symmetry (multiplicity 2, Wyckoff symbol *a*). It is surrounded by eight Si1 atoms (4*mm*, 4 *e*) and eight Cu1/Fe1 atoms (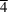
*m*2, 4 *d*), forming the centre of a tetra­deca­hedron. The shortest distance between calcium and silicon is Ca1—Si1 = 3.087 (4) Å, whereas the longest Ca1—Cu1/Fe1 bond is 3.216 (2) Å.

This study refined the crystal structure model of CaCu_1.424_Fe_0.576_Si_2_based on single-crystal X-ray diffraction data. Its com­position was confirmed by EDX results.

## Synthesis and crystallization

High-purity elements Ca (99.5% purity, 0.068 g), Cu (99.5% purity, 0.4718 g), Fe (99.9% purity, 0.0247 g) and Si (99.5% purity, 0.4270 g) were weighed precisely according to a stoichiometric ratio of 7:30:2:61. The mixture was homogenized and thoroughly ground in an agate mortar. Subsequently, the blended powder was loaded into a tungsten carbide die with a 5 mm inner diameter and com­pacted at 6 MPa for 3 min to form cylindrical pellets. These pellets were subjected to high-pressure sinter­ing experiments using a six-anvil apparatus (Liu & Fan, 2018 ▸), where samples were pressurized to 6 GPa and heated to 1573 K for 40 min, followed by rapid quenching to room tem­per­a­ture through furnace power termination. A regular specimen was selected and mounted on a glass fiber using adhesive for X-ray diffraction measurements.

## Refinement

Comprehensive crystallographic data, data collection parameters and structure refinement details are summarized in Table 1 ▸. To facilitate com­parative analysis, the labelling scheme and atomic coordinates for CaCu_1.424_Fe_0.576_Si_2_ were taken from the corresponding data of CaCu_2_Si_2_ (Palenzona *et al.*, 1986 ▸) and CaFe_2_Si_2_ (Hlukhyy *et al.*, 2012 ▸). The sites of the occupancy factors for the co-occupancy of the Cu and Fe atoms refined to 0.71 (15) and 0.29 (15), respectively. The command ‘SHEL 999 0.84’ was used to eliminate weakly diffracting high-angle data. The maximum and mini­mum residual electron densities in the final difference map are located at 0.99 Å from Ca1 and 0.00 Å from Cu1, respectively.

## Supplementary Material

Crystal structure: contains datablock(s) I. DOI: 10.1107/S2414314625003256/bt4168sup1.cif

Structure factors: contains datablock(s) I. DOI: 10.1107/S2414314625003256/bt4168Isup2.hkl

supplementary file. DOI: 10.1107/S2414314625003256/bt4168sup3.docx

CCDC reference: 2442775

Additional supporting information:  crystallographic information; 3D view; checkCIF report

## Figures and Tables

**Table 1:** Experimental details

Crystal data	
Chemical formula	CaCu_1.42_Fe_0.58_Si_2_
*M*_r_	218.91
Crystal system, space group	Tetragonal, *I*4/*mmm*
Temperature (K)	296
*a*, *c* (Å)	4.041 (3), 10.010 (9)
*V* (Å^3^)	163.5 (3)
*Z*	2
Radiation type	Mo *K*α
μ (mm^−1^)	13.82
Crystal size (mm)	0.10 × 0.07 × 0.06

Data collection	
Diffractometer	Bruker D8 Venture Photon 100 CMOS
Absorption correction	Multi-scan (*SADABS*; Krause *et al.*, 2015 ▸)
*T*_min_, *T*_max_	0.383, 0.746
No. of measured, independent and observed [*I* > 2σ(*I*)] reflections	707, 60, 46
*R*_int_	0.097
(sin θ/λ)_max_ (Å^−1^)	0.588

Refinement	
*R*[*F*^2^ > 2σ(*F*^2^)], *wR*(*F*^2^), *S*	0.071, 0.156, 1.29
No. of reflections	60
No. of parameters	9
Δρ_max_, Δρ_min_ (e Å^−3^)	1.05, −1.45

Computer programs: *APEX5* (Bruker, 2023 ▸), *SAINT* (Bruker, 2023 ▸), *SHELXT* (Sheldrick, 2015*a* ▸), *SHELXL* (Sheldrick, 2015*b* ▸), *DIAMOND* (Brandenburg & Putz, 2017 ▸) and *publCIF* (Westrip, 2010 ▸).

## full crystallographic data

### Calcium copper iron disilicide Crystal data

**Table:**

CaCu_1.42_Fe_0.58_Si_2_	*D*_x_ = 4.448 Mg m^−^^3^
*M**_r_* = 218.91	Mo *K*α radiation, λ = 0.71073 Å
Tetragonal, *I*4/*m**m**m*	Cell parameters from 452 reflections
*a* = 4.041 (3) Å	θ = 4.1–26.9°
*c* = 10.010 (9) Å	µ = 13.82 mm^−^^1^
*V* = 163.5 (3) Å^3^	*T* = 296 K
*Z* = 2	Lump, gray
*F*(000) = 209	0.10 × 0.07 × 0.06 mm

### Calcium copper iron disilicide Data collection

**Table:**

Bruker D8 Venture Photon 100 CMOS diffractometer	46 reflections with *I* > 2σ(*I*)
phi and ω scans	*R*_int_ = 0.097
Absorption correction: multi-scan (SADABS; Krause *et al.*, 2015)	θ_max_ = 24.7°, θ_min_ = 4.1°
*T*_min_ = 0.383, *T*_max_ = 0.746	*h* = −4→4
707 measured reflections	*k* = −4→4
60 independent reflections	*l* = −11→11

### Calcium copper iron disilicide Refinement

**Table:**

Refinement on *F*^2^	9 parameters
Least-squares matrix: full	0 restraints
*R*[*F*^2^ > 2σ(*F*^2^)] = 0.071	*w* = 1/[σ^2^(*F*_o_^2^) + (0.0957*P*)^2^] where *P* = (*F*_o_^2^ + 2*F*_c_^2^)/3
*wR*(*F*^2^) = 0.156	(Δ/σ)_max_ < 0.001
*S* = 1.29	Δρ_max_ = 1.05 e Å^−^^3^
60 reflections	Δρ_min_ = −1.45 e Å^−^^3^

### Calcium copper iron disilicide Special details

**Table:**

Geometry. All esds (except the esd in the dihedral angle between two l.s. planes) are estimated using the full covariance matrix. The cell esds are taken into account individually in the estimation of esds in distances, angles and torsion angles; correlations between esds in cell parameters are only used when they are defined by crystal symmetry. An approximate (isotropic) treatment of cell esds is used for estimating esds involving l.s. planes.

### Calcium copper iron disilicide Fractional atomic coordinates and isotropic or equivalent isotropic displacement parameters (Å^2^)

**Table:**

	*x*	*y*	*z*	*U*_iso_*/*U*_eq_	Occ. (<1)
Fe1	0.000000	0.500000	0.250000	0.0171 (18)	0.29 (15)
Cu1	0.000000	0.500000	0.250000	0.0171 (18)	0.71 (15)
Si1	0.000000	0.000000	0.3834 (10)	0.011 (3)	
Ca1	0.000000	0.000000	0.000000	0.011 (3)	

### Calcium copper iron disilicide Atomic displacement parameters (Å^2^)

**Table:**

	*U*^11^	*U*^22^	*U*^33^	*U*^12^	*U*^13^	*U*^23^
Fe1	0.0178 (19)	0.0178 (19)	0.016 (3)	0.000	0.000	0.000
Cu1	0.0178 (19)	0.0178 (19)	0.016 (3)	0.000	0.000	0.000
Si1	0.013 (4)	0.013 (4)	0.006 (7)	0.000	0.000	0.000
Ca1	0.009 (4)	0.009 (4)	0.013 (7)	0.000	0.000	0.000

### Calcium copper iron disilicide Geometric parameters (Å, º)

**Table:**

Fe1—Si1	2.422 (6)	Cu1—Si1^i^	2.422 (6)
Fe1—Si1^i^	2.422 (6)	Cu1—Si1^ii^	2.422 (6)
Fe1—Si1^ii^	2.422 (6)	Cu1—Si1^iii^	2.422 (6)
Fe1—Si1^iii^	2.422 (6)	Cu1—Ca1^vi^	3.216 (2)
Fe1—Fe1^i^	2.857 (2)	Cu1—Ca1	3.216 (2)
Fe1—Fe1^iv^	2.857 (2)	Cu1—Ca1^ii^	3.216 (2)
Fe1—Fe1^iii^	2.857 (2)	Cu1—Ca1^vii^	3.216 (2)
Fe1—Fe1^v^	2.857 (2)	Si1—Si1^viii^	2.33 (2)
Fe1—Ca1^vi^	3.216 (2)	Si1—Ca1^vi^	3.087 (4)
Fe1—Ca1	3.216 (2)	Si1—Ca1^ix^	3.087 (4)
Fe1—Ca1^ii^	3.216 (2)	Si1—Ca1^x^	3.087 (4)
Fe1—Ca1^vii^	3.216 (2)	Si1—Ca1^vii^	3.087 (4)
Cu1—Si1	2.422 (6)		
			
Si1—Fe1—Si1^i^	107.7 (2)	Fe1^i^—Si1—Fe1	72.3 (2)
Si1—Fe1—Si1^ii^	113.1 (4)	Fe1^xi^—Si1—Fe1	113.1 (4)
Si1^i^—Fe1—Si1^ii^	107.7 (2)	Si1^viii^—Si1—Cu1	123.5 (2)
Si1—Fe1—Si1^iii^	107.7 (2)	Si1^viii^—Si1—Fe1^iii^	123.5 (2)
Si1^i^—Fe1—Si1^iii^	113.1 (4)	Fe1^i^—Si1—Fe1^iii^	113.1 (4)
Si1^ii^—Fe1—Si1^iii^	107.7 (2)	Fe1^xi^—Si1—Fe1^iii^	72.3 (2)
Si1—Fe1—Fe1^i^	53.85 (10)	Fe1—Si1—Fe1^iii^	72.3 (2)
Si1^i^—Fe1—Fe1^i^	53.85 (10)	Si1^viii^—Si1—Ca1^vi^	67.78 (18)
Si1^ii^—Fe1—Fe1^i^	126.15 (10)	Fe1^i^—Si1—Ca1^vi^	138.99 (9)
Si1^iii^—Fe1—Fe1^i^	126.15 (10)	Fe1^xi^—Si1—Ca1^vi^	138.99 (9)
Si1—Fe1—Fe1^iv^	126.15 (10)	Fe1—Si1—Ca1^vi^	70.27 (4)
Si1^i^—Fe1—Fe1^iv^	126.15 (10)	Cu1—Si1—Ca1^vi^	70.27 (4)
Si1^ii^—Fe1—Fe1^iv^	53.85 (10)	Fe1^iii^—Si1—Ca1^vi^	70.27 (4)
Si1^iii^—Fe1—Fe1^iv^	53.85 (10)	Si1^viii^—Si1—Ca1^ix^	67.78 (18)
Fe1^i^—Fe1—Fe1^iv^	180.0	Fe1^i^—Si1—Ca1^ix^	70.27 (4)
Si1—Fe1—Fe1^iii^	53.85 (10)	Fe1^xi^—Si1—Ca1^ix^	70.27 (4)
Si1^i^—Fe1—Fe1^iii^	126.15 (10)	Fe1—Si1—Ca1^ix^	138.99 (9)
Si1^ii^—Fe1—Fe1^iii^	126.15 (10)	Fe1^iii^—Si1—Ca1^ix^	138.99 (9)
Si1^iii^—Fe1—Fe1^iii^	53.85 (10)	Ca1^vi^—Si1—Ca1^ix^	135.6 (4)
Fe1^i^—Fe1—Fe1^iii^	90.0	Si1^viii^—Si1—Ca1^x^	67.78 (18)
Fe1^iv^—Fe1—Fe1^iii^	90.0	Fe1^i^—Si1—Ca1^x^	138.99 (9)
Si1—Fe1—Fe1^v^	126.15 (10)	Fe1^xi^—Si1—Ca1^x^	70.27 (4)
Si1^i^—Fe1—Fe1^v^	53.85 (10)	Fe1—Si1—Ca1^x^	138.99 (9)
Si1^ii^—Fe1—Fe1^v^	53.85 (10)	Fe1^iii^—Si1—Ca1^x^	70.27 (4)
Si1^iii^—Fe1—Fe1^v^	126.15 (10)	Ca1^vi^—Si1—Ca1^x^	81.78 (13)
Fe1^i^—Fe1—Fe1^v^	90.0	Ca1^ix^—Si1—Ca1^x^	81.78 (13)
Fe1^iv^—Fe1—Fe1^v^	90.0	Si1^viii^—Si1—Ca1^vii^	67.78 (18)
Fe1^iii^—Fe1—Fe1^v^	180.0	Fe1^i^—Si1—Ca1^vii^	70.27 (4)
Si1—Fe1—Ca1^vi^	64.60 (15)	Fe1^xi^—Si1—Ca1^vii^	138.99 (9)
Si1^i^—Fe1—Ca1^vi^	162.4 (2)	Fe1—Si1—Ca1^vii^	70.27 (4)
Si1^ii^—Fe1—Ca1^vi^	64.60 (15)	Fe1^iii^—Si1—Ca1^vii^	138.99 (9)
Si1^iii^—Fe1—Ca1^vi^	84.5 (2)	Ca1^vi^—Si1—Ca1^vii^	81.78 (13)
Fe1^i^—Fe1—Ca1^vi^	116.37 (2)	Ca1^ix^—Si1—Ca1^vii^	81.78 (13)
Fe1^iv^—Fe1—Ca1^vi^	63.628 (19)	Ca1^x^—Si1—Ca1^vii^	135.6 (4)
Fe1^iii^—Fe1—Ca1^vi^	63.63 (2)	Si1^iii^—Ca1—Si1^xii^	180.0
Fe1^v^—Fe1—Ca1^vi^	116.37 (2)	Si1^iii^—Ca1—Si1^xiii^	135.6 (4)
Si1—Fe1—Ca1	84.5 (2)	Si1^xii^—Ca1—Si1^xiii^	44.4 (4)
Si1^i^—Fe1—Ca1	64.60 (15)	Si1^iii^—Ca1—Si1^xiv^	44.4 (4)
Si1^ii^—Fe1—Ca1	162.4 (2)	Si1^xii^—Ca1—Si1^xiv^	135.6 (4)
Si1^iii^—Fe1—Ca1	64.60 (15)	Si1^xiii^—Ca1—Si1^xiv^	180.0
Fe1^i^—Fe1—Ca1	63.63 (2)	Si1^iii^—Ca1—Si1^i^	81.78 (13)
Fe1^iv^—Fe1—Ca1	116.37 (2)	Si1^xii^—Ca1—Si1^i^	98.22 (13)
Fe1^iii^—Fe1—Ca1	63.63 (2)	Si1^xiii^—Ca1—Si1^i^	81.78 (13)
Fe1^v^—Fe1—Ca1	116.37 (2)	Si1^xiv^—Ca1—Si1^i^	98.22 (13)
Ca1^vi^—Fe1—Ca1	127.26 (4)	Si1^iii^—Ca1—Si1^xv^	81.78 (13)
Si1—Fe1—Ca1^ii^	162.4 (2)	Si1^xii^—Ca1—Si1^xv^	98.22 (13)
Si1^i^—Fe1—Ca1^ii^	64.60 (15)	Si1^xiii^—Ca1—Si1^xv^	81.78 (13)
Si1^ii^—Fe1—Ca1^ii^	84.5 (2)	Si1^xiv^—Ca1—Si1^xv^	98.22 (13)
Si1^iii^—Fe1—Ca1^ii^	64.60 (15)	Si1^i^—Ca1—Si1^xv^	135.6 (4)
Fe1^i^—Fe1—Ca1^ii^	116.37 (2)	Si1^iii^—Ca1—Si1^xvi^	98.22 (13)
Fe1^iv^—Fe1—Ca1^ii^	63.63 (2)	Si1^xii^—Ca1—Si1^xvi^	81.78 (13)
Fe1^iii^—Fe1—Ca1^ii^	116.37 (2)	Si1^xiii^—Ca1—Si1^xvi^	98.22 (13)
Fe1^v^—Fe1—Ca1^ii^	63.63 (2)	Si1^xiv^—Ca1—Si1^xvi^	81.78 (13)
Ca1^vi^—Fe1—Ca1^ii^	127.26 (4)	Si1^i^—Ca1—Si1^xvi^	44.4 (4)
Ca1—Fe1—Ca1^ii^	77.83 (7)	Si1^xv^—Ca1—Si1^xvi^	180.0
Si1—Fe1—Ca1^vii^	64.60 (15)	Si1^iii^—Ca1—Si1^xvii^	98.22 (13)
Si1^i^—Fe1—Ca1^vii^	84.5 (2)	Si1^xii^—Ca1—Si1^xvii^	81.78 (13)
Si1^ii^—Fe1—Ca1^vii^	64.60 (15)	Si1^xiii^—Ca1—Si1^xvii^	98.22 (13)
Si1^iii^—Fe1—Ca1^vii^	162.4 (2)	Si1^xiv^—Ca1—Si1^xvii^	81.78 (13)
Fe1^i^—Fe1—Ca1^vii^	63.63 (2)	Si1^i^—Ca1—Si1^xvii^	180.0
Fe1^iv^—Fe1—Ca1^vii^	116.37 (2)	Si1^xv^—Ca1—Si1^xvii^	44.4 (4)
Fe1^iii^—Fe1—Ca1^vii^	116.37 (2)	Si1^xvi^—Ca1—Si1^xvii^	135.6 (4)
Fe1^v^—Fe1—Ca1^vii^	63.63 (2)	Si1^iii^—Ca1—Fe1	45.14 (14)
Ca1^vi^—Fe1—Ca1^vii^	77.83 (7)	Si1^xii^—Ca1—Fe1	134.86 (14)
Ca1—Fe1—Ca1^vii^	127.26 (4)	Si1^xiii^—Ca1—Fe1	96.72 (17)
Ca1^ii^—Fe1—Ca1^vii^	127.26 (4)	Si1^xiv^—Ca1—Fe1	83.28 (17)
Si1—Cu1—Si1^i^	107.7 (2)	Si1^i^—Ca1—Fe1	45.14 (14)
Si1—Cu1—Si1^ii^	113.1 (4)	Si1^xv^—Ca1—Fe1	96.72 (17)
Si1^i^—Cu1—Si1^ii^	107.7 (2)	Si1^xvi^—Ca1—Fe1	83.28 (17)
Si1—Cu1—Si1^iii^	107.7 (2)	Si1^xvii^—Ca1—Fe1	134.86 (14)
Si1^i^—Cu1—Si1^iii^	113.1 (4)	Si1^iii^—Ca1—Fe1^iii^	45.14 (14)
Si1^ii^—Cu1—Si1^iii^	107.7 (2)	Si1^xii^—Ca1—Fe1^iii^	134.86 (14)
Si1—Cu1—Ca1^vi^	64.60 (15)	Si1^xiii^—Ca1—Fe1^iii^	96.72 (17)
Si1^i^—Cu1—Ca1^vi^	162.4 (2)	Si1^xiv^—Ca1—Fe1^iii^	83.28 (17)
Si1^ii^—Cu1—Ca1^vi^	64.60 (15)	Si1^i^—Ca1—Fe1^iii^	96.72 (17)
Si1^iii^—Cu1—Ca1^vi^	84.5 (2)	Si1^xv^—Ca1—Fe1^iii^	45.14 (14)
Si1—Cu1—Ca1	84.5 (2)	Si1^xvi^—Ca1—Fe1^iii^	134.86 (14)
Si1^i^—Cu1—Ca1	64.60 (15)	Si1^xvii^—Ca1—Fe1^iii^	83.28 (17)
Si1^ii^—Cu1—Ca1	162.4 (2)	Fe1—Ca1—Fe1^iii^	52.74 (4)
Si1^iii^—Cu1—Ca1	64.60 (15)	Si1^iii^—Ca1—Cu1	45.14 (14)
Ca1^vi^—Cu1—Ca1	127.26 (4)	Si1^xii^—Ca1—Cu1	134.86 (14)
Si1—Cu1—Ca1^ii^	162.4 (2)	Si1^xiii^—Ca1—Cu1	96.72 (17)
Si1^i^—Cu1—Ca1^ii^	64.60 (15)	Si1^xiv^—Ca1—Cu1	83.28 (17)
Si1^ii^—Cu1—Ca1^ii^	84.5 (2)	Si1^i^—Ca1—Cu1	45.14 (14)
Si1^iii^—Cu1—Ca1^ii^	64.60 (15)	Si1^xv^—Ca1—Cu1	96.72 (17)
Ca1^vi^—Cu1—Ca1^ii^	127.26 (4)	Si1^xvi^—Ca1—Cu1	83.28 (17)
Ca1—Cu1—Ca1^ii^	77.83 (7)	Si1^xvii^—Ca1—Cu1	134.86 (14)
Si1—Cu1—Ca1^vii^	64.60 (15)	Si1^iii^—Ca1—Fe1^xviii^	134.86 (14)
Si1^i^—Cu1—Ca1^vii^	84.5 (2)	Si1^xii^—Ca1—Fe1^xviii^	45.14 (14)
Si1^ii^—Cu1—Ca1^vii^	64.60 (15)	Si1^xiii^—Ca1—Fe1^xviii^	83.28 (17)
Si1^iii^—Cu1—Ca1^vii^	162.4 (2)	Si1^xiv^—Ca1—Fe1^xviii^	96.72 (17)
Ca1^vi^—Cu1—Ca1^vii^	77.83 (7)	Si1^i^—Ca1—Fe1^xviii^	134.86 (14)
Ca1—Cu1—Ca1^vii^	127.26 (4)	Si1^xv^—Ca1—Fe1^xviii^	83.28 (17)
Ca1^ii^—Cu1—Ca1^vii^	127.26 (4)	Si1^xvi^—Ca1—Fe1^xviii^	96.72 (17)
Si1^viii^—Si1—Fe1^i^	123.5 (2)	Si1^xvii^—Ca1—Fe1^xviii^	45.14 (14)
Si1^viii^—Si1—Fe1^xi^	123.5 (2)	Fe1—Ca1—Fe1^xviii^	180.0
Fe1^i^—Si1—Fe1^xi^	72.3 (2)	Fe1^iii^—Ca1—Fe1^xviii^	127.26 (4)
Si1^viii^—Si1—Fe1	123.5 (2)		

Symmetry codes: (i) −*x*−1/2, −*y*+1/2, −*z*+1/2; (ii) *x*, *y*+1, *z*; (iii) −*x*+1/2, −*y*+1/2, −*z*+1/2; (iv) −*x*+1/2, −*y*+3/2, −*z*+1/2; (v) −*x*−1/2, −*y*+3/2, −*z*+1/2; (vi) *x*+1/2, *y*+1/2, *z*+1/2; (vii) *x*−1/2, *y*+1/2, *z*+1/2; (viii) −*x*, −*y*, −*z*+1; (ix) *x*−1/2, *y*−1/2, *z*+1/2; (x) *x*+1/2, *y*−1/2, *z*+1/2; (xi) *x*, *y*−1, *z*; (xii) *x*−1/2, *y*−1/2, *z*−1/2; (xiii) −*x*−1/2, −*y*−1/2, −*z*+1/2; (xiv) *x*+1/2, *y*+1/2, *z*−1/2; (xv) −*x*+1/2, −*y*−1/2, −*z*+1/2; (xvi) *x*−1/2, *y*+1/2, *z*−1/2; (xvii) *x*+1/2, *y*−1/2, *z*−1/2; (xviii) −*x*, −*y*, −*z*.
