# Supplementary material for: CaCu1.424Fe0.576Si2
Source: IUCrdata. 2025 Apr 17;10(Pt 4):x250325. doi: 10.1107/S2414314625003256 (PMC12054748; doi:10.1107/S2414314625003256)
Supplement: Supplementary file 3 [file x-10-x250325-sup3.docx]

**SUPPLEMENTARY MATERIALS:**

**Crystal structure of CaCu_1.42_Fe_0.58_Si_2_**

Yangkun Dai^a^, Yibo Liu^a^, Marek Mihalkovič^b^,Bin Wen^a^, Lifeng Zhang^ac^, and Changzeng Fan^a,d^*

^a^ State Key Laboratory of Metastable Materials Science and Technology, Yanshan University,

Qinhuangdao 066004, People’s Republic of China

^b^ Institute of Physics, Slovak Academy of Sciences, 84511 Bratislava, Slovakia

^c^ School of Mechanical and Materials Engineering, North China University of Technology, Beijing, People’s Republic of China

^d^ Hebei Key Lab for Optimizing Metal Product Technology and Performance, Yanshan University, Qinhuangdao, Hebei 066004, People’s Republic of China

*Correspondence email: [chzfan@ysu.edu.cn](mailto:chzfan@ysu.edu.cn)

The chemical compositions were examined quantitatively by energy dispersive X-ray spectroscopy (EDX) analysis attached to a Hitachi S-3400N SEM for the purpose of guiding the crystal structure refinement. The examined points and areas are designated in Fig. S1, and the corresponding results are listed in Table S1. The deviation relative to the results of refinement of chemical composition is probably caused by the tilt of the single crystal surface to the incident beam. In addition, the conductive adhesives and glues may also result in the detected impurity elements of carbon. However, the presence of all elements of this phase in some parts of the scan proves the existence of this phase. For ease of reading, the atomic ratio of Ca, Si, Cu, Fe and C was calculated and shown in the last column of Table S1.


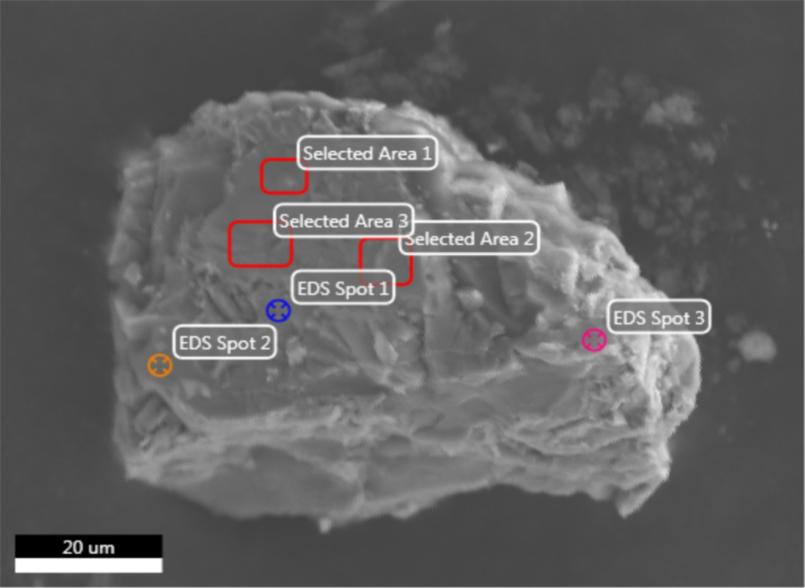
Fig. S1 Single crystal of CaCu_1.42_Fe_0.58_Si_2_ with selected spots and areas for EDX analysis

**Table S1 EDX results for selected points and areas as designated in Fig. S1**

|  | Element | Weight (%) | Atomic (%) | Error (%) |
| --- | --- | --- | --- | --- |
| Spot1 | C | 10.46 | 33.35 | 11.50 |
|  | Si | 11.68 | 15.93 | 7.14 |
|  | Ca | 6.35 | 6.07 | 3.48 |
|  | Fe | 1.11 | 0.76 | 10.82 |
|  | Cu | 69.58 | 41.94 | 2.25 |
| Spot2 | C | 14.27 | 36.93 | 11.18 |
|  | Si | 20.72 | 22.94 | 5.89 |
|  | Ca | 17.55 | 13.61 | 2.68 |
|  | Fe | 0.18 | 0.10 | 60.56 |
|  | Cu | 45.02 | 22.03 | 2.47 |
| Spot3 | Si | 49.21 | 66.77 | 5.19 |
|  | Ca | 1.75 | 1.67 | 7.46 |
|  | Fe | 26.06 | 17.78 | 2.60 |
|  | Cu | 22.97 | 13.78 | 3.22 |
| Area1 | C | 10.05 | 29.26 | 11.73 |
|  | Si | 20.96 | 26.11 | 6.27 |
|  | Ca | 13.57 | 11.84 | 2.95 |
|  | Fe | 0.18 | 0.11 | 61.01 |
|  | Cu | 53.85 | 29.64 | 2.41 |
| Area2 | C | 24.68 | 53.97 | 10.24 |
|  | Si | 13.70 | 12.81 | 5.93 |
|  | Ca | 13.43 | 8.80 | 2.60 |
|  | Fe | 0.14 | 0.07 | 61.45 |
|  | Cu | 44.40 | 18.36 | 2.42 |
| Area3 | C | 15.24 | 41.22 | 11.11 |
|  | Si | 15.89 | 18.38 | 6.46 |
|  | Ca | 10.32 | 8.37 | 3.12 |
|  | Fe | 0.24 | 0.14 | 59.69 |
|  | Cu | 56.93 | 29.11 | 2.37 |
